# ANCA negative pauci-immune glomerulonephritis with systemic involvement

**DOI:** 10.4103/0971-4065.62096

**Published:** 2010

**Authors:** K. Sampathkumar, M. Ramakrishnan, A. K. Sah, S. Gowtham, R. N. Ajeshkumar

**Affiliations:** Department of Nephrology, Meenakshi Mission Hospital and Research Centre, Madurai - 625 107, India

**Keywords:** Anti-neutrophil cytoplasmic antibody, rapidly progressive glomerulonephritis, renal failure

## Abstract

Systemic vasculitides (SV) are a group of diseases with multi system involvement and varied clinical presentation. Anti-neutrophil cytoplasmic antibody (ANCA) testing has high sensitivity and specificity for SV. We describe the clinical course of four patients who had pauci-immune glomerulonephritis with systemic involvement without serological ANCA positivity; they were followed up for a cumulative 55 patient months. The mean Birmingham vasculitis score score was 23. All four had systemic symptoms with arthralgias and fever (100%). Neurological manifestations were seen in two patients (66%). Accelerated hypertension was seen in one. One patient had pulmonary renal syndrome. Renal manifestation was characterized by nephrotic range of proteinuria with glomerular hematuria in all (100%) and severe renal failure requiring dialysis in three (66%). At admission the mean blood urea was 146 ± 19 mg% and mean serum creatinine was 5.6 ± 1.9 mg%. Renal biopsy revealed focal proliferative glomerulonephritis with crescents only in 20-30% of glomeruli. There was significant chronic interstitial involvement in two patients (66%). Therapy with pulse steroids, cyclophosphamide, and mycophenolate mofetil (MMF) was effective in three patients while one died with lung hemorrhage. In conclusion, majority of patients with ANCA negative pauci-immune glomerulonephritis have multi-system involvement at admission. Renal biopsy is characterized by focal proliferative lesions with crescents and significant chronic interstitial fibrosis. Immunosuppressive drugs in the form of corticosteroids, MMF and cyclophosphamide bring about marked renal recovery in most patients.

## Introduction

Pauci-immune crescentic necrotizing glomerulonephritis (GN) is defined clinically by rapidly progressive glomerulonephritis and histologically by the presence of focal glomerular necrosis and extracapillary proliferation in the absence of significant glomerular immune deposits. In 1982, Davies *et al*. described for the first time autoantibodies directed against neutrophil cyctoplasmic targets.[1] Later Van der Waude *et al*. published their work in Wegener's granulomatosis, wherein auto antibodies were described against ethanol fixed neutrophils.[2] This was followed by the work of Falk and Jennette on patients with microscopic polyangitis, in whom perinuclear staining pattern of auto antibodies was described.[3] The presence of either PR3-anti-neutrophil cytoplasmic antibody (ANCA) (proteinase 3) or MPO-ANCA (Myeloperoxidase) proved in a meta analysis by Rao *et al*. and an European study by Hagen *et al*. to have a sensitivity of 91% and specificity of 98% for active pauci immune vasculitis/glomerulonephritis.[4,5] In 10% of the cases with systemic vasculitis, ANCA is negative. Only few case series describe the clinical profile of this group. Here we discuss the clinical presentation of four cases of systemic vasculitis with negative serology for ANCA. Disease activity at initial clinical presentation was evaluated by using the Birmingham vasculitis assessment score (BVAS).[6] This scoring system consists of a list of items that are based on clinical history and physical examination supported by laboratory data of patients with systemic vasculitis.

## Case Reports

### Case 1

A 62-year-old male presented with hematuria, oliguria, and arthralgia of three weeks duration with renal impairment. He had a renal biopsy done one week ago elsewhere, which revealed mild mesangial proliferation with acute tubular necrosis. He was started on hemodialysis. Serological testing was not done at that time. There was no recovery after four weeks, following which he was referred here. Two days prior to admission he developed seizures with fever. At admission his blood pressure was 160/100 mm Hg. Serology for auto antibodies was negative as shown in Table 1. Serum complement levels were normal. He was treated by hemodialysis and a repeat renal biopsy was performed in our center which showed focal glomerular tuft infiltration by neutrophils. 20% of the glomeruli showed crescents. IF was negative for immunoglobulin deposits. It is likely that the initial biopsy had missed the glomerular crescents due to their focal nature. He was treated with three intravenous methyl prednisolone, each of 1 gram pulse, followed by oral cyclophosphamide therapy in a dose of 2 mg/kg/day which was continued for 12 weeks. His renal failure resolved after four weeks. The patient was switched to mycophenolate mofetil (MMF) at a dose of 1.5 gram/d. When he attained a sustained clinical remission the dose of MMF was reduced to 500 mg once a day. However, he developed a relapse twelve months later with renal failure requiring dialysis. MMF was again restored to 1.5 gm per day and his renal function improved after two weeks. He is currently on follow-up with serum creatinine level of 1.3 mg% with normal urinalysis.

**Table 1:** Clinical, laboratory, renal biopsy and follow-up data

	Case 1	Case 2	Case 3	Case 4
Age	62	52	7	36
Sex	Male	Male	Female	Male
Fever	+	+	+	+
Arthralgia	+	+	+	+
Hemoptysis	−	+	−	−
Neurological	Seizures	−	Seizures, Bell's palsy	−
Blood pressure	160/100	150/90	210/120	150/100
Chest X-ray	Transient infiltrates in mid lung zones.	Multiple cavities in both lung fields [Figure 1]	−	Normal
Urinalysis	Protein 3+, 10–12 RBC's/hpf	Protein 4+, 20–25 RBCs/hpf	Protein 3+, 10–12 RBCs/hpf	Protein 3+, 10–12 RBC's
Urine protein creatinine ratio	5.2:1.0	15.2: 1.0	7:1	5.5:1
ESR	120 mm/hr	80 mm/hr	110/hr	75 mm/hr
Serum C3,C4	Normal	Normal	Normal	N
Blood urea/creatinine mg%	145/7.6	167/5.4	128/3.8	184/10
BVAS score	23/63	31/63	24/63	15/63
Renal biopsy	LM:10 glomeruli, one obsolescent. Focal proliferative lesions in four. Two showed cellular crescents. Interstitial fibrosis 21 IF: Negative	LM:8 glomeruli with mesangial proliferation. Four showed cellular crescents. Interstitial fibrosis 1+ IF: Negative	LM:7 glomeruli. Two obsolescent. Focal necrosis with crescents in two glomerulus. Interstitial fibrosis-3+ IF: Negative [Figure 2]	LM:29 glomeruli 26 showed crescents (89%) focal fibrinoid necrosis. Normal interstitium
Follow-up	36 months. current S. Cr. − 1.3 mg.	4 weeks. Died due to pulmonary hemorrhage	16 months. Current S. Cr. − 1.8 mg.	8 weeks. Current S.Cr. −2.4 mg%

LM: Light microscopy; S. Cr.: Serum creatinine; BUAS: Birmingham vasculitis assessment score

### Case 2

**Figure 1:**
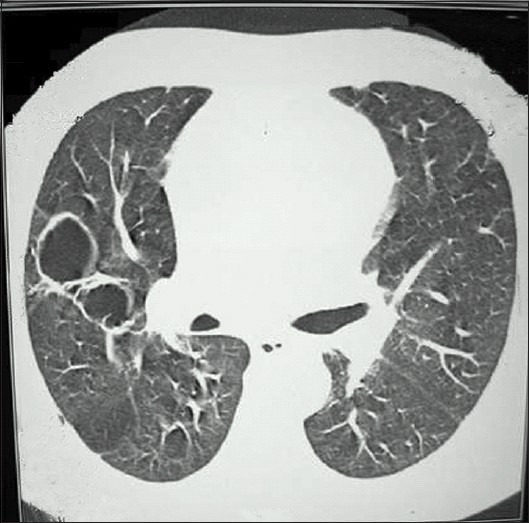
Multiple pulmonary cavities in case 2. The patient succumbed to massive hemoptysis

A-52-year-old man presented with two weeks history of fever associated with arthralgia, cough with recurrent hemoptysis, and oliguria. Chest X-ray showed multiple thin walled cavities in both lungs [Figure 1]. Investigations are shown in Table 1. Serology for auto antibodies was negative as shown. Renal biopsy showed pauciimmune crescentic glomerulonephritis with 50% crescents. The IF was negative for immunoglobulin deposits. He was treated with pulse methyl prednisolone and oral cyclophosphamide. He died within four weeks of admission with severe pulmonary hemorrhage.

### Case 3

**Figure 2:**
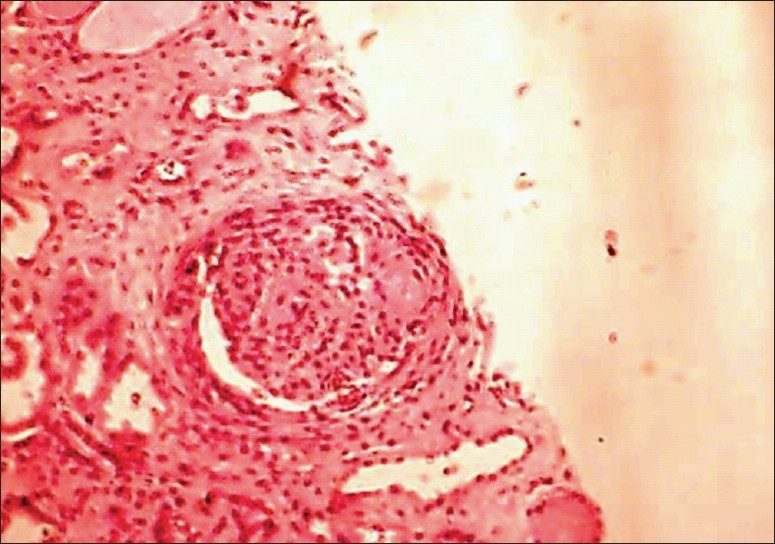
Renal biopsy of case 3 showing glomerular crescent and focal necrosis. Areas of interstitial fibrosis are seen

A seven-year-old girl was admitted with two weeks history of fever, headache, and puffiness of face. Physical findings revealed severe hypertension (210/120 mm Hg) with left sided Bell's palsy. Investigations showed proteinuria, glomerular hematuria, and renal failure [Table 1]. Serology for auto antibodies was negative as shown in Table 1. Serum complement levels were normal. Renal biopsy showed significant chronic tubulointerstitial changes with focal crescents. The IF was negative for immunoglobulin deposits [Figure 2]. She was started on pulse methyl prednisolone along with cyclophosphamide, which was switched over to mycophenolate mofetil after eight weeks. Her renal function has stabilized with a serum creatinine level of 1.8 mg. However, she requires four different classes of anti hypertensive drugs for control of blood pressure. Currently, she is on follow-up for the past 16 months.

### Case 4

A 36-year-old male was admitted with one month history of fatigue, fever, and arthralgias, which was followed by oliguria for one week. His blood pressure at admission was 150/100 mm Hg. Urinalysis showed high protein creatinine ratio with glomerular hematuria. He was in severe renal failure and underwent hemodialysis. Serology for auto antibodies was negative as shown in Table 1. Serum complement levels were normal. A percutaneous renal biopsy revealed 89% of the glomeruli had cellular crescents and focal fibrinoid necrosis with intact tubulointerstitium. The IF was negative for immunoglobulin deposits. He was started on pulse intravenous methyl prednisolone and cyclophosphamide.After eight weeks his renal function improved and is creatinine is currently 2.4 mg%. His current immunosuppression consists of MMF (1 G/day) along with 0.5 mg/kg/day of prednisolone.

## Discussion

ANCA are antibodies to human neutrophil cytoplasmic granule proteins. There are two main types of ANCA pattern that can be discerned on the basis of indirect immunofluorescence (IIF) on ethanol-fixed human neutrophils, namely cytoplasmic or cANCA staining, and perinuclear or pANCA staining. The main antigens involved the staining patterns are protease 3 (PR3), which closely correlates with the cANCA Indirect Immunoflourescense pattern, and myeloperoxidase (MPO), which is responsible for the majority of pANCA patterns. International consensus guidelines recommend all sera are first tested by IIF, and subsequently by ELISA for PR3 and MPO if the IIF is positive. We tested our patients for IIF followed by anti MPO and PR-3 antibodies and they were negative. There is a wealth of literature on the ANCA positive systemic vasculitis with regards to clinical presentation, pathogenesis, pathology and treatment. But, approximately 10% of systemic vasculitis patients test negative for ANCA. This group has been studied only infrequently. Hedger *et al*. investigated 35 patients with ANCA-negative rapidly progressive glomerulonepritis and found that they had fewer airway symptoms than ANCA-positive ones but failed to find any other difference between these two subgroups of patients.[7] In a multicentric retrospective observational study Eisenberger *et al*. have identified 20 cases with ANCA negative vasculitis.[8] The renal histology revealed a high percentage of active glomerular lesions (50%), mainly cellular crescents, whereas only 28% of them had glomerular necrosis. Chronic tissue damage with glomerulosclerosis (21%) and diffuse interstitial fibrosis (40%) were already present at diagnosis, more prominent than in historical ANCA positive patients. This has been our observation also that chronic interstitial fibrosis is already present even though the history of the clinical illness is relatively short.

Another recent study from China analyzed patients with ANCA negative vasculitis.[9] Clinical and pathologic characteristics were compared between patients with and without ANCA. Among the 85 patients with pauci-immune GN, 28 (32.9%) were ANCA negative. Compared with the 57 ANCA-positive patients, the ANCA-negative patients were much younger. The level of urinary protein and the prevalence of nephrotic syndrome were significantly higher in ANCA-negative patients than that in ANCA-positive patients. This has been our observation also that nephrotic range of proteinuria is common in ANSV. However, the prevalence of extrarenal involvement was significantly lower in ANCA-negative patients. The renal pathology was more severe in ANCA negative groups and the renal survival was poorer.

At variance with the published series, our patients had prominent extra renal manifestations in the form of arthralgia, fever, seizures, mono neuritis and lung involvement. The first case presented with predominant renal involvement. The second case had pulmonary renal syndrome while the third case had renal and facial nerve involvement. Renal biopsy showed pauciimmune state with cellular crescents in all the cases. Glomeruli showed focal proliferative lesions with neutrophilic infiltrates. However necrotic lesions in the glomeruli were rare. There was a significant interstitial fibrosis in two cases. Response to immunosuppressive treatment was excellent in three patients while the one with pulmonary involvement succumbed to the disease. Among the systemic vasculitides Classic polyarteritis nodosa (PAN) patients do not have circulating ANCA. However, classic PAN commonly presents with renal infarction with minor degrees of proteinuria due to medium vessel involvement. In contrast our patients had nephrotic range of proteinuria with crescents. Churg-Strauss syndrome has the lowest positivity rate for ANCA (< 5% for PR3-ANCA and 40% for MPO-ANCA).[10,11] However, none of our patients had eosinophilia or tissue eosinophil infiltrates.

Age has important bearing on the prognosis of ANCA negative systemic vasculitis. In Ute Eisenberger's series age above 65 years was associated with high mortality. The age group in our series is considerably younger with none of the patients above 65 years of age. Secondly, our patients have fared better due to the fact that MMF was used more often. MMF is increasingly being found useful in the induction and remission phases of systemic vasculitides.[12] In another study 32 consecutive patients with 34 episodes of active vasculitis who could not be treated with cyclophosphamide were followed up after starting therapy with MMF. Complete remission (CR) was obtained in 25 (78%) patients, partial remission (PR) in 6 (19%), whereas 1 (3%) patient did not respond.[13] In a study comparing cyclophosphamide to MMF in Chinese patients with paucimmune vasculitis and moderate renal involvement, it was shown that MMF was superior in inducing remission (77% versus 47% in cyclophosphamide group). 44% of MMF treated group recovered normal renal function against 15% in the cyclophosphamide group.[14]

It is becoming increasingly clear by human and experimental data that ANCAs are not only diagnostic markers but pathogenetically linked to vasculitic process too. Described briefly, neutrophils are primed by TNF-alpha and express cell surface MPO and PR-3Subsequent interaction with ANCA leads on to endothelial damage and transmigration of neutrophils and their degranulation with resultant necrotizing vasculitis.[15] Anti-endothelial cell antibodies (AECA) have been implicated to have a causal role in ANCA negative vasculitis. In a study involving sera from 19 patients with ANCA negative glomerulonephritis, 10 were positive for AECA. In ANCA-negative pauci-immune GN, 10 of 19 patients were serum IgG-AECA positive and seven bands reactive with endothelial antigens could be blotted. The prevalence of skin rash and thrombocytosis was significantly higher in patients with anti-76 kDa and anti-123 kDa AECA autoantibodies than in patients without, respectively. BVAS of patients with anti-200 kDa AECA were significantly higher than in patients without ANCA positivity.[16] Another newly investigated marker is Circulating Angiopoietin-2 which is elevated and closely correlates with BVAS in systemic vasculitides with renal involvement. Angiopoietin-2 might be a potential mediator of endothelial cell detachment.[17] An intrinsic podocyte defect may also be operative. In a conditional knock out mouse model specific podocyte deletion of VHL gene (Von Hippel-Lindau gene) has been shown to lead on to crescentic glomerulonephritis without ANCA positivity. VHL protein product interacts with hypoxia-inducible factor 1 (HIF-1) whose targets include tumour necrosis factor-a (TNF-α), vascular endothelial growth factor-A (VEGF-A), the chemokine receptor 4 (CXC-R4) all of which are implicated in the glomerular damage due to vasculitis.[18]

## Conclusion

Patients who present with ANCA negative vasculitis with pauci immune glomerulonephritis have significant systemic involvement. Renal lesions are characterized by focal proliferation with crescents associated with chronic tubulointerstitial changes. Immunosuppressive drugs in the form of corticosteroids, cyclophosphamide, and MMF bring about significant improvement in prognosis. Further research is needed to elucidate the pathogenesis and focus on specific diagnostic markers for this group.
